# Supplementary material for: Smooth Interpolating Curves with Local Control and Monotone Alternating Curvature
Source: Comput Graph Forum. 2022 Oct 6;41(5):25–38. doi: 10.1111/cgf.14600 (PMC9827861; doi:10.1111/cgf.14600)
Supplement: Supplementary file 1 — Supplement Material [file CGF-41-25-s001.zip › Local-Smooth-Interpolating-MonoCurvature/extern/clothoids/submodules/quarticRootsFlocke/docs/api-cpp/class_a00035.html]

Class Quartic — Quartic Roots v1.0.0

### Navigation

- index
- toc
- next
- previous
- Quartic Roots »
- C++ API »
- Class Quartic

# Class Quartic¶

- Defined in File PolynomialRoots.hh

## Class Documentation¶

class PolynomialRoots::Quartic¶
:   Quartic polynomial class

    **Constructor**

    ```
    double a = 1;
    double b = 2;
    double c = 3;
    double d = 3;
    double e = 3;
    Quartic p(a,b,c,d,e); // build an solve ``a x^4 + b x^3 + c x^2 + d x + e = 0``


    Quartic p;
    p.setup(a,b,c,d,e); // build an solve ``a x^4 + b x^3 + c x^2 + d x + e = 0``
    ```

    **Get kind of solution**

    ```
    int nroots = p.numRoots(); 
    int nroots = p.numRealRoots();
    int nroots = p.numComplexRoots();
    ```

    **Get real roots**

    ```
    double r_min = 0;
    double r_max = 2;
    double r[4];
    int nroots;
    nroots = p.getRealRoots( r );
    nroots = p.getPositiveRoots( r );
    nroots = p.getNegativeRoots( r );
    nroots = p.getRootsInRange( r_min, r_max, r );
    nroots = p.getRootsInOpenRange( r_min, r_max, r );
    ```

    **Get roots**

    ```
    double r0 = p.real_root0();
    double r1 = p.real_root1();
    double r2 = p.real_root2();
    double r3 = p.real_root3();
    complexType r0 = p.root0();
    complexType r1 = p.root1();
    complexType r2 = p.root2();
    complexType r3 = p.root3();

    complexType r;
    double re, im;
    p.getRoot0( re, im );
    p.getRoot0( r );
    p.getRoot1( re, im );
    p.getRoot1( r );
    p.getRoot2( re, im );
    p.getRoot2( r );
    p.getRoot3( re, im );
    p.getRoot3( r );
    ```

    **Evaluate polynomial**

    ```
    {double or complex} v, x; 
    v = p.eval( x );

    p.eval( x, p, dp );
    ```

    **Information**

    ```
    p.info( cout );
    bool ok = p.check( cout );
    ```

    Public Functions

    inline Quartic()¶

    inline Quartic(valueType \_a, valueType \_b, valueType \_c, valueType \_d, valueType \_e)¶

    inline void setup(valueType a, valueType b, valueType c, valueType d, valueType e)¶
    :   Compute the roots of quartic polynomial \( a x^4 + b x^3 + c x^2 + d x + e \)

        Parameters
        :   - **a** – **[in]** coefficient of \( x^4 \)
            - **b** – **[in]** coefficient of \( x^3 \)
            - **c** – **[in]** coefficient of \( x^2 \)
            - **d** – **[in]** coefficient of \( x \)
            - **e** – **[in]** coefficient of \( x^0 \)

    inline indexType numRoots() const¶
    :   Number of roots found.

    inline indexType numRealRoots() const¶
    :   Number of real roots.

    inline indexType numComplexRoots() const¶
    :   Number of complex roots

    indexType getRealRoots(valueType r[]) const¶
    :   Get the real roots.

        Parameters
        :   **r** – **[out]** vector that will be filled with the real roots

        Returns
        :   the total number of real roots, 0, 1, 2, 3 or 4

    indexType getPositiveRoots(valueType r[]) const¶
    :   Get positive real roots

        Parameters
        :   **r** – **[out]** vector that will be filled with the real roots

        Returns
        :   the total number of positive real roots, 0, 1, 2, 3 or 4

    indexType getNegativeRoots(valueType r[]) const¶
    :   Get negative real roots.

        Parameters
        :   **r** – **[out]** vector that will be filled with the real roots

        Returns
        :   the total number of negative real roots, 0, 1, 2, 3 or 4

    indexType getRootsInRange(valueType a, valueType b, valueType r[]) const¶
    :   Get real roots in a closed range.

        Parameters
        :   - **a** – **[in]** left side of the range
            - **b** – **[in]** right side of the range
            - **r** – **[out]** vector that will be filled with the real roots

        Returns
        :   the total number of real roots in the range [a,b]

    indexType getRootsInOpenRange(valueType a, valueType b, valueType r[]) const¶
    :   Get real roots in an open range.

        Parameters
        :   - **a** – **[in]** left side of the range
            - **b** – **[in]** right side of the range
            - **r** – **[out]** vector that will be filled with the real roots

        Returns
        :   the total number of real roots in the open range (a,b)

    inline valueType real\_root0() const¶
    :   First real root.

    inline valueType real\_root1() const¶
    :   Second real root.

    inline valueType real\_root2() const¶
    :   Third real root.

    inline valueType real\_root3() const¶
    :   Fourth real root.

    inline complexType root0() const¶
    :   First real or complex root.

    inline complexType root1() const¶
    :   Second real or complex root.

    inline complexType root2() const¶
    :   Third real or complex root.

    inline complexType root3() const¶
    :   4th real or complex root.

    inline void getRoot0(valueType &re, valueType &im) const¶
    :   First real or complex root.

    inline void getRoot0(complexType &r) const¶
    :   First real or complex root.

    inline void getRoot1(valueType &re, valueType &im) const¶
    :   Second real or complex root.

    inline void getRoot1(complexType &r) const¶
    :   Second real or complex root.

    inline void getRoot2(valueType &re, valueType &im) const¶
    :   Third real or complex root.

    inline void getRoot2(complexType &r) const¶
    :   Third real or complex root.

    inline void getRoot3(valueType &re, valueType &im) const¶
    :   4th real or complex root.

    inline void getRoot3(complexType &r) const¶
    :   4th real or complex root.

    inline valueType eval(valueType x) const¶
    :   Evalute the quartic polynomial.

        Parameters
        :   **x** – value where compute \( p(x) \), x complex

        Returns
        :   the value \( p(x) \)

    inline complexType eval(complexType const &x) const¶
    :   Evalute the quartic polynomial.

        Parameters
        :   **x** – value where compute \( p(x) \), x complex

        Returns
        :   the value \( p(x) \)

    void info(std::ostream &s) const¶
    :   Print info of the roots of the polynomial.

    bool check(std::ostream &s) const¶
    :   Check tolerenace and quality of the computed roots.

### Quick search

### Table of Contents

- Quartic Roots Solver
- C++ API

«
hide menu

menu
sidebar
»

### Navigation

- index
- toc
- next
- previous
- Quartic Roots »
- C++ API »
- Class Quartic

© Copyright 2021, Enrico Bertolazzi.
Created using Sphinx 4.2.0.
